# Group B *Streptococcus* Sequence Type 103 as Human and Bovine Pathogen, Brazil

**DOI:** 10.3201/eid3008.231575

**Published:** 2024-08

**Authors:** Laura M.A. Oliveira, Leandro C. Simões, Chiara Crestani, Natália S. Costa, José Carlos F. Pantoja, Renata F. Rabello, Sérgio E.L. Fracalanzza, Lucia M. Teixeira, Uzma B. Khan, Dorota Jamrozy, Stephen Bentley, Tatiana C.A. Pinto, Ruth N. Zadoks

**Affiliations:** Federal University of Rio de Janeiro, Rio de Janeiro, Brazil (L.M.A. Oliveira, L.C. Simões, N.S. Costa, S.E.L. Fracalanzza, L.M. Teixeira, T.C.A. Pinto);; Pasteur Institut, Paris, France (C. Crestani);; Universidade Estadual Paulista Júlio de Mesquita Filho, São Paulo, Brazil (J.C.F. Pantoja);; Fluminense Federal University, Rio de Janeiro (R.F. Rabello);; Wellcome Sanger Institute, Hinxton, UK (U.B. Khan, D. Jamrozy, S. Bentley);; University of Sydney, Camden, New South Wales, Australia (R.N. Zadoks)

**Keywords:** streptococci, bacteria, zoonoses, *Streptococcus agalactiae*, Group B *Streptococcus*, bovine mastitis, molecular epidemiology, cattle, humans, Brazil

## Abstract

Group B *Streptococcus* sequence type 103 is known primarily as a bovine mastitis pathogen. In Brazil, it has circulated in cattle and humans since the 1990s. It lacks *scp*B and, in humans, was found only among carriage isolates. Bovine–human interspecies transmission may have contributed to its evolution and spread.

Group B *Streptococcus* (GBS) is a major cause of life-threatening neonatal infections and bovine mastitis (*1*). The GBS population is composed of host-specialist lineages, such as sequence type (ST) 17, and host-generalist lineages, such as ST23 (*2*). ST103 is common among bovine GBS (bGBS) in Europe (*3*,*4*), Colombia (*5*), and China (*6*); reports of human GBS (hGBS) ST103 are rare and mostly limited to Asia (*7*–*9*). This difference may reflect limitations to host or geographic range or may be attributable to surveillance efforts. We investigated the molecular epidemiology of GBS STs in humans and cattle in Brazil.

## The Study

We extracted genomic DNA from hGBS (carriage n = 416, disease n = 39) and bGBS (milk n = 151, environment n = 5) isolates collected in Brazil during 1978–2021 (Table 1) by using the DNeasy Blood & Tissue Kit (QIAGEN, https://www.qiagen.com). We generated whole-genome sequences at the Wellcome Sanger Institute (Hixton, UK; https://www.gbsgen.net) or at MicrobesNG (Birmingham, UK; https://microbesng.com) by using the Illumina NovaSeq platform (https://www.illumina.com). We used the sequences to predict ST, capsular types, antimicrobial resistance (AMR), and surface protein profiles (Alpha, Alp1, Alp2/3, Rib, Srr1, Srr2, pilus islands PI1, PI-2A1, PI-2A2, and PI-2B, and HvgA) with GBS Typer version 1.0.11 (https://github.com/sanger-bentley-group/GBS-Typer-sanger-nf), and to detect *scp*B gene carriage by using BLASTn (https://blast.ncbi.nlm.nih.gov) and GenBank reference sequence AF327852.1. We performed whole-genome alignment by using Snippy v4.6.0 (https://github.com/tseemann/snippy) and constructed a phylogenetic tree with RAxML as implemented in Gubbins version 3.3.1 (https://github.com/nickjcroucher/gubbins).

**Table 1:** Origin of GBS isolates recovered from human and bovine samples submitted to whole-genome sequencing, Brazil*

Host	Clinical source	Region	Collection year	ST103 GBS	Non-ST103 GBS
Human†	Anovaginal carriage‡	Rio de Janeiro	1979–2021	14	329
	Oropharynx§	Rio de Janeiro	1978–2014	2	8
	Umbilical swab	Rio de Janeiro	2001	0	1
	Semen	Rio de Janeiro	2017–2018	1	61
	Urine	Rio de Janeiro	1990–2019	0	16
		São Paulo	2009	0	3
		Porto Alegre	2006	0	5
		Cuiabá	2009	0	2
	Invasive disease specimens¶	Rio de Janeiro	1990–2021	0	13
Bovine	Milk	Rio de Janeiro#	1987–2007	17	38
		Minas Gerais**	1996–2021	6	51
		São Paulo††	1987–2021	23	16
	Farm environment	Minas Gerais‡‡	2010	4	1

*GBS, group B *Streptococcus*; ST, sequence type. †Human isolates were epidemiologically unrelated. ‡Anovaginal carriage: anovaginal specimens collected from pregnant women between the 35th and 37th gestational weeks during routine antenatal care. §Oropharynx: throat swab. ¶Invasive disease specimens: blood, cerebrospinal fluid, cerebrospinal fluid, bronchoalveolar lavage, peritoneal fluid, bone, abscess. #Isolates represent 18 farms and 53 cows. **Isolates represent 21 farms and 46 cows. ††Isolates represent 6 farms and 21 cows. ‡‡Isolates represent 1 farm and 5 environmental samples.

**Figure 1:**
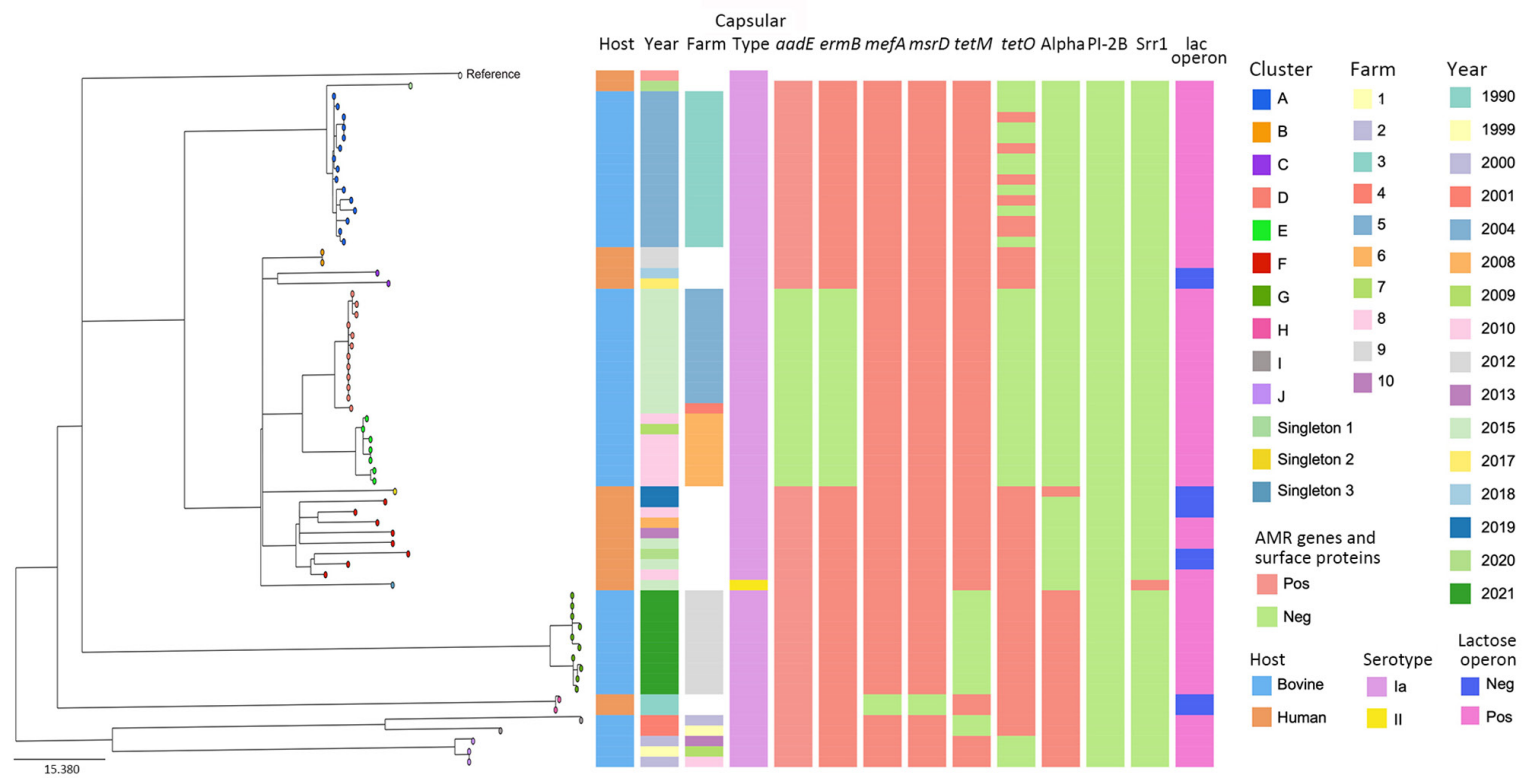
Phylogeny of group B *Streptococcus* isolates belonging to ST103 recovered from cattle and human populations in Brazil, 1990‒2021. The tree was built based on single-nucleotide polymorphisms extracted from an alignment outside recombination regions, created by mapping reads of each isolate to the sequence of the ST103 reference strain GBS85147 (GenBank accession no. CP010319.1). Scale bar indicates substitutions per site. AMR, antimicrobial resistance; neg, negative; pos, positive; ST, sequence type.

We included only GBS isolates assigned to ST103 in this study. Of the 611 isolates tested, 67 (11%) isolates belonged to ST103 (17 hGBS isolates, 1990–2020; 50 bGBS, 1999–2021) (Table 2), which showed that ST103 hGBS circulated in Brazil in parallel with bGBS well before it was reported elsewhere (*7*–*9*). In accordance with previous studies (*2*,*4*), ST103 isolates mostly belonged to serotype Ia, with 1 exception (hGBS, serotype II), which was also the only *ssr*1-negative isolate (Figure 1). Although rare, capsular switching can occur in GBS and may impair the effectiveness of future polysaccharide vaccines (*10*).

**Table 2:** Distribution of group B *Streptococcus* ST103 isolates from human and bovine samples, Brazil*

Host	Sample	No. isolates	Accession nos. (ENA/GenBank)
Human	Anovaginal	14	ERR9738291†, ERR9738561†, ERR9738563†, ERR9738359†, ERR9738596†, ERR9738481, ERR9738404†‡, ERR9738442†, ERR9738474, ERR9738564, ERR9738631, ERR9738643, ERR9937783†, ERR9937793
	Oropharynx	2	ERR9738674, ERR9738675
	Semen	1	ERR9738366
Bovine	Milk	46	ERR9738322, ERR9937866, ERR9937886, ERR9937890, ERR9937910, ERR9937940, ERR9738322, ERR9937792, ERR9937904, ERR9937944, ERR9937946, ERR9937948, ERR9937950, ERR9937952, ERR9937954, ERR9937956, ERR9937966, ERR9937968, ERR9937970, ERR9937972, ERR9937974, ERR9937976, ERR9937978, ERR9937980, ERR9937936, ERR9937703, ERR9937937, ERR9937705, ERR9937707, ERR9937939, BioProject ID: PRJNA1086968 (JBBHGR000000000, JBBHGQ000000000, JBBHGP000000000, JBBHGO000000000, JBBHGN000000000, JBBHGM000000000, JBBHGL000000000, JBBHGK000000000, JBBHGJ000000000, JBBHGI000000000, JBBHGH000000000, JBBHGG000000000, JBBHGF000000000, JBBHGE000000000, JBBHGD000000000, JBBHGC000000000)
	Farm environment	4	ERR9937891, ERR9937699, ERR9937892, ERR9937893

*ST103 identified from 619 human and bovine isolates collected in Brazil during 1978–2021. All ST103 isolates were negative for *scpB*. All bovine isolates were lactose fermenting, as were 8 human isolates from anovaginal samples. All but 1 isolate belonged to serotype Ia. ENA, European Nucleotide Archive; ST, sequence type. †Human isolates positive for lactose fermentation. ‡Human isolate belonging to serotype II.

The core-genome phylogeny showed 10 clusters, largely representing either host or, for bGBS, farm of origin, and 3 singletons (Figure 1). Some bGBS (clusters D and E) were more closely related to hGBS (clusters B, C, and F) than to other bGBS (clusters G, I, and J) (Figure 1). The hGBS were distributed across 4 clusters and 3 singletons. Clusters B (n = 2 isolates), C (n = 2), and F (n = 8) formed a clade with bGBS clusters D and E and were detected throughout the period studied (1990–2020) (Figure 1). Cluster H (n = 2), which is located on a long branch in the phylogeny, included 2 historical strains isolated in 1990 from human oropharynx samples.

Some accessory genome traits were consistent across the entire phylogeny, whereas others differed between or within host species. All isolates carried pilus island PI-2B, as previously decribed (*2*,*4*). All hGBS and bGBS isolates lacked the *scp*B gene, which encodes C5a peptidase and is crucial for GBS adhesion and invasion of human cells (*11*). The absence of *scp*B gene may explain why ST103 was only found among carriage isolates in humans, although a larger sample size or in vitro studies would be needed to provide statistical or mechanistic support for that hypothesis.

The Alpha protein gene was absent in clusters nearest to the tree root, regardless of host, year or, for bGBS, farm of isolation, suggesting that the element was acquired later by the diverged subpopulation (clusters A–F). By contrast, the lactose operon was consistently present in all bGBS but not in all hGBS, suggesting either multiple loss or acquisition events in those host-associated clusters. The ability to ferment lactose, as mediated by the Lac.2 operon, drives GBS adaptation to the bovine udder (*2*). Presence of Lac.2 and the phenotypic ability to ferment lactose are relatively rare in human isolates (*4*,*12*). In our study, all bGBS and almost half of hGBS (n = 8, 47%) were able to ferment lactose, suggesting bovine-to-human transmission with subsequent loss of Lac.2 in the absence of selective pressure for its maintenance in the human niche. This finding contrasts with an earlier study, which suggested a human origin of bovine ST103 (*3*). In-depth analysis of the evolution of this clade using a larger collection of isolates across a broad geographic range would be needed to determine the timing and direction of host jumps.

**Figure 2:**
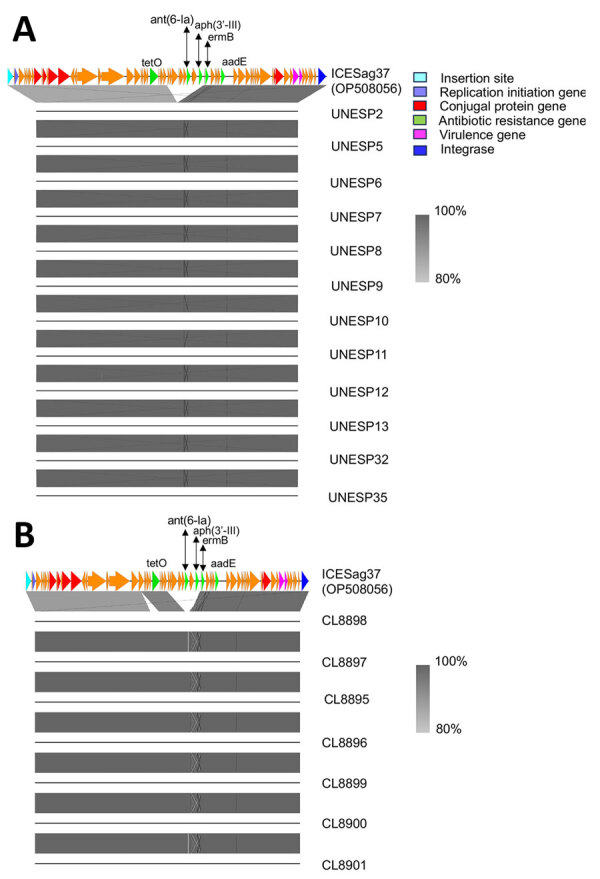
Alignment of nucleotide sequences of bovine group B *Streptococcus* isolates belonging to ST103 collected in Brazil with ICESag37, an integrative conjugative element associated with antimicrobial resistance and virulence genes. A) Sequence alignment for cluster D isolates (n = 12). B) Sequence alignment for cluster E isolates (n = 7). The analysis used Easyfig version 2.2 to perform BLASTn (https://blast.ncbi.nlm.nih.gov) comparisons between isolates in clusters D and E against the reference ICEsag37 (GenBank accession no. OP508056). Arrows indicate mobility genes and conjugal transfer proteins (red), *rep*A initiator genes (purple), critical site-specific recombinase (blue), antibiotic resistance genes (green), and other ICE genes (orange). Aminoglycoside genes normally found in ICESag37 (*ant*(6–1a) and *aph*(3-III)) are missing from the bovine isolates.

We observed the greatest variability in accessory genome content in AMR genes. In bGBS, *tet*O was the most common resistance gene, followed by *tet*M, but never in combination. In hGBS, AMR genes were rarely found (Figure 1); this finding is in contrast to many other human-associated GBS lineages, which have a high prevalence of tetracycline resistance genes (*13*). In addition to *tet*O, bGBS clusters D and E also carried *erm*B and *aad*E on a variant of ICESag37 (OP508056), an integrative conjugative element associated with AMR and virulence genes, with a median nucleotide similarity of 86% (Figure 2). Overall, prevalence of AMR genes was higher in bGBS than in hGBS (88% vs. 17.6% of isolates carrying >1 AMR gene). Sampling bias might have contributed to this finding; several herds were represented by multiple isolates, which formed monophyletic clusters with homogeneous AMR profiles, as expected based on contagious transmission of bGBS within dairy herds. Nevertheless, we detected AMR in bGBS in every single herd, and AMR prevalence in bGBS would still be higher than in hGBS if each herd was represented by a single isolate, indicating a true biologic effect. The high prevalence of AMR among bGBS may be driven by the overuse of antimicrobial drugs in dairy herds in emerging economies like that of Brazil (*1*), where macrolides, tetracycline, and aminoglycosides are among the most commonly used antimicrobial drugs for treatment and prevention of clinical mastitis (*14*).

Vaccination is desirable to prevent hGBS and bGBS disease without reliance on antimicrobial drugs, but no human or bovine vaccines are licensed yet. Promising candidates for a human maternal vaccine include both polysaccharide-protein conjugate and protein subunit strategies. Surveillance of capsular types and surface proteins of emerging GBS lineages, including in low- and middle-income countries, is crucial to inform vaccine design, which can impair vaccine effectiveness. In our study, all ST103 strains were associated with serotype Ia and PI-2B, Srr1, and Alpha surface proteins, which are among the most immunogenic vaccine targets (*15*).

## Conclusions

GBS is a multihost pathogen, able to adapt to different niches. The phylogeny of ST103 and the presence of the lactose operon in hGBS suggest that interspecies transmission (bovine-to-human) might have contributed to the evolution of ST103 in Brazil. In addition, our results suggest that the presence of the pilus variant PI-2B and absence of the *scp*B gene are common markers of ST103 in Brazil, irrespective of host species. The lack of *scp*B may limit the virulence of GBS ST103 in humans, which in turn may have contributed to its underreporting, especially in countries or studies that focus on clinical isolates. Additional studies at population and mechanistic level would be needed to fully understand the origin, evolution, epidemiology, and virulence potential of this emerging clade, but our results show that ST103 is more common in hGBS than previously described and that it has been circulating in Brazil at least since the 1990s. 
